# *GSTP1* c.313A > G mutation is an independent risk factor for neutropenia hematotoxicity induced by anthracycline-/paclitaxel-based chemotherapy in breast cancer patients

**DOI:** 10.1186/s12957-022-02679-y

**Published:** 2022-06-22

**Authors:** Juanzi Zeng, Heming Wu, Donghua Liu, Liang Li, Jiaquan Li, Qiuming Wang, Min Ye, Qingyan Huang, Zhikang Yu, Jinfeng Zhang

**Affiliations:** 1 Department of Medical Oncology, Meizhou People’s Hospital (Huangtang Hospital), Meizhou Academy of Medical Sciences, Meizhou, People’s Republic of China; 2 Guangdong Provincial Key Laboratory of Precision Medicine and Clinical Translational Research of Hakka Population, Meizhou People’s Hospital (Huangtang Hospital), Meizhou Academy of Medical Sciences, Meizhou, People’s Republic of China; 3 Center for Precision Medicine, Meizhou People’s Hospital (Huangtang Hospital), Meizhou Academy of Medical Sciences, Meizhou, People’s Republic of China

**Keywords:** Breast cancer, *GSTP1*, Polymorphism, Chemotherapy toxicity

## Abstract

**Background:**

The link between glutathione S-transferase P1 (GSTP1) c.313A > G polymorphism and chemotherapy-related adverse events remains controversial. The goal of this study was to assess how this variant affected the toxicity of anthracycline-/paclitaxel-based chemotherapy in patients with breast cancer.

**Methods:**

This study retrospectively investigated pharmacogenetic associations of *GSTP1* c.313A > G with chemotherapy-related adverse events in 142 breast cancer patients who received anthracycline and/or paclitaxel chemotherapy.

**Results:**

There were 61 (43.0%), 81 (57.0%), 43 (30.3%), and 99 (69.7%) patients in the T0-T2, T3-T4, N0-N1, and N2-N3 stages, respectively. There were 108 (76.1%) patients in clinical stages I–III and 34 (23.9%) patients in clinical stage IV. The numbers of patients with luminal A, luminal B, HER2 + , and triple-negative breast cancer (TNBC) were 10 (7.0%), 77 (54.2%), 33 (23.2%), and 22 (15.5%), respectively. The numbers of patients who carried *GSTP1* c.313A > G A/A, A/G, and G/G genotypes were 94 (66.2%), 45 (31.7%), and 3 (2.1%), respectively. There were no statistically significant differences in the proportion of certain toxicities in patients with A/G, G/G, and A/G + G/G genotypes, except for neutropenia, in which the proportion of patients with A/G + G/G (*χ*^2^ = 6.586, *P* = 0.035) genotypes was significantly higher than that with the AA genotype. The logistic regression analysis indicated that *GSTP1* c.313A > G mutation (A/G + G/G vs. A/A genotype) (adjusted OR 4.273, 95% CI 1.141–16.000, *P* = 0.031) was an independent variable associated with neutropenia.

**Conclusions:**

The findings of this study indicate that the *GSTP1* c.313A > G mutation is an independent risk factor for neutropenia hematotoxicity in breast cancer patients induced by anthracycline-/paclitaxel-based chemotherapy.

## Introduction

Breast cancer is a malignant tumor that develops in the breast’s epithelial tissue, and the majority of sufferers are women [1]. Breast cancer is the most commonly diagnosed cancer in women worldwide, and it is also the leading cause of cancer death in women [2]. Because China has such huge population, women are increasingly stressed at work and in their personal lives, and the annual growth rate of breast cancer has surpassed the global norm [3]. There are several factors that can increase the risk of breast cancer, such as gender, age, estrogen, family history, unhealthy lifestyle, and genetic variations [4].

According to hormone receptors (HRs) (including estrogen receptor (ER) and progesterone receptor (PR)), human epidermal growth factor receptor 2 (HER2), and Ki67 (a proliferation index marker) status, breast cancer is classified into four major subgroups, including luminal A, luminal B, HER2-enriched (HER2 +), and triple-negative breast cancer (TNBC) subtypes [5]. Luminal A subtype breast cancer is defined as ER-positive (ER +), PR ≥ 20%, HER2-negative (HER2-), and Ki67 < 20%. luminal B-like (HER2-) breast cancer is ER + , HER2 − , Ki67 ≥ 20%, and PR < 20%. luminal B-like (HER2 +) breast cancer is ER + , HER2 + , any Ki67 level, and PR level. Luminal B-like (HER2-) and luminal B-like (HER2 +) are collectively called luminal B type. HER2 + subtype breast cancer is defined as HER2 + , ER − , and PR − . TNBC is defined as ER − , PR − , and HER2 −  [6, 7]. Distinct molecular kinds of breast cancer have different therapies, effectiveness, and recurrence risks [8, 9].

In recent years, precision therapy has received increasing attention. Breast cancer treatment has evolved into a mature system that includes cytotoxic chemotherapy, molecularly targeted therapy, endocrine therapy, and immunotherapy [10]. Cytotoxic chemotherapy is still one of the most common treatments for breast cancer. Chemotherapy is an important part of the comprehensive treatment of breast cancer. Based on different application periods, it is classified as postoperative adjuvant chemotherapy for early breast cancer, preoperative neoadjuvant chemotherapy for early or locally advanced breast cancer, first-line, and multiline rescue chemotherapy for advanced breast cancer [11]. Anthracycline and paclitaxel drugs are the cornerstones of breast cancer chemotherapy and are widely used in all of the above treatment stages [12]. Anthracycline- and paclitaxel-based chemotherapy is one of the primary established treatment options for breast cancer [13].

In clinical practice, patients with the same tumor stage, pathological type, and treatment regimen experience varying degrees of adverse reactions after treatment with anthracycline- and paclitaxel-based chemotherapy. It may be related to the patient’s clinical characteristics, environmental factors, and genetic factors [14, 15]. Some studies showed that the metabolism of cytotoxic chemotherapy drugs in vivo is affected by glutathione S-transferases (GSTs) [16, 17]. GSTs are II-phase metabolic enzymes found in the human body that are involved in the metabolism of xenobiotic compounds and their reactive products, prevent oxidative stress, and catalyze the combination of electrophilic substances and reduced glutathione to exert detoxification effects. The metabolic activation of both anthracycline and paclitaxel is catalyzed by the GSTs during liver metabolism [18]. Mutations in the *GSTP1* gene may increase the sensitivity of chemotherapy drugs to cells by decreasing the activity of the GSTP1 enzyme and the body’s ability to metabolize and excrete chemotherapy drugs [19]. The *GSTP1* c.313A > G variant (Ile105Val, rs1695) may reduce the activity of the GSTP1 enzyme, which is a widely concerned polymorphism and the most studied mutation site of the *GSTP1* gene at present [20, 21].

Although there have been several studies on the relationship between *GSTP1* gene polymorphisms and chemotherapy toxicity in breast cancer, the findings are controversial, particularly in different populations [22, 23]. The goal of our study is to look into the link between *GSTP1* gene polymorphisms and adverse reactions to anthracycline-/paclitaxel-based chemotherapy in breast cancer patients from the Meizhou Hakka ethnic group in southern China. We performed a systematic retrospective study in a cohort of 142 Meizhou Hakka breast cancer patients.

## Materials and methods

### Participants

This retrospective clinical study included 142 patients with breast cancer who visited Meizhou People’s Hospital (Huangtang Hospital) between September 2016 and September 2019. The following were the study subjects’ inclusion criteria: (1) patients with histopathologically confirmed breast cancer; (2) patients who received cytotoxic chemotherapy based on anthracycline-/paclitaxel-based chemotherapy agents; (3) patients with no serious liver, kidney, or heart diseases; and (4) patients who were above the age of 18. The following were the study subjects’ exclusion criteria: (1) patients with tumors other than breast cancer; (2) patients with severe liver, kidney, or heart disease insufficiency before treatment; and (3) other circumstances inconsistent with the inclusion criteria mentioned above. The Ethics Committee of the Meizhou People’s Hospital approved this study, which was conducted in accordance with the Declaration of Helsinki.

### Chemotherapy regimens and toxicity evaluation

Patients received anthracycline-/paclitaxel-based cytotoxic chemotherapy according to the following regimens:

TEC regimen: docetaxel (T) (75 mg/m^2^), epirubicin (E) (75 mg/m^2^) and cyclophosphamide (C) (500 mg/m^2^).

EC-T regimen: epirubicin (E) (90 mg/m^2^) and cyclophosphamide (C) (600 mg/m^2^); followed by docetaxel (T) (90 mg/m^2^).

EC-TH regimen: epirubicin (E) (90 mg/m^2^) and cyclophosphamide (C) (600 mg/m^2^); followed by docetaxel (T) (90 mg/m^2^) and trastuzumab (H) (initial dose 8 mg/kg, followed by 6 mg/kg).

EC regimen: epirubicin (E) (90 mg/m^2^) and cyclophosphamide (C) (600 mg/m^2^).

TCbH regimen: taxane (T) (175 mg/m^2^), carboplatin (Cb) [area under curve (AUC) of 6], and trastuzumab (H) (initial dose 8 mg/kg, followed by 6 mg/kg).

TCb regimen: taxane (T) (175 mg/m^2^) and carboplatin (Cb) [area under curve (AUC) of 6].

A total of 114 patients were treated with the TEC regimen, 11 patients with the EC-T regimen, 12 patients with the EC-TH regimen, 3 patients with the TCbH regimen, and 1 patient with TCb, and EC regimen, respectively (Table 1). All drugs were injected intravenously and chemotherapy was administered once every 3 weeks over the course of, at least 2 cycles. All patients in this study were given standard drug doses of different regimens in the first course of treatment, and drug regimens and dosages in subsequent treatment cycles were adjusted according to the efficacy. During treatment, pay close attention to the side effects of drugs on patients. Erythropoiesis stimulating agents (ESAs) and thrombopoietin should be used to ameliorate symptoms of anemia and thrombocytopenia caused by the myelosuppression of chemotherapy drugs, and blood transfusion should be used if necessary. Granulocyte colony-stimulating factor (G-CSF) was not used of prophylaxis for neutropenia in the first course of treatment. G-CSF should be used only in subsequent cycles when grade 3 or higher neutropenia is present. Blood samples were collected to detect liver function indexes of patients before each cycle of medication. If abnormal liver function occurred, hepato-protective agents were given. If the patient has nausea, vomiting, diarrhea, constipation, and other gastrointestinal adverse reactions, symptomatic treatment should be given.

**Table 1:** Clinical characteristics of breast cancer patients

	Number (mean ± SD)	Percentage (%)
Age (year)		
<35	16 (31.00 ± 2.68)	11.3
35–50	60 (43.20 ± 4.70)	42.2
>50	66 (57.47 ± 5.19)	46.5
Menopause		
No	83	58.5
Yes	59	41.5
T-stage		
T0–T2	61	43.0
T3–T4	81	57.0
N-stage		
N0–N1	43	30.3
N2–N3	99	69.7
Clinical stage		
I–III	108	76.1
IV	34	23.9
Molecular subtypes		
Luminal A	10	7.0
Luminal B	77	54.2
HER2 +	33	23.2
TNBC	22	15.5
Chemotherapy regimen^a^		
TEC (docetaxel 75 mg/m^2^, epirubicin 75 mg/m^2^)	114	80.3
EC-T/EC-TH (docetaxel 90 mg/m^2^, epirubicin 90 mg/m^2^)	23	16.2
TCbH (taxane 175 mg/m^2^)	3	2.1
TCb (taxane 175 mg/m^2^)	1	0.7
EC (epirubicin 90 mg/m^2^)	1	0.7

*ER* Estrogen receptor, *PR* Progesterone receptor; *TNBC* Triple-negative breast cancer

^a^Only the doses of anthracycline and paclitaxel in different regimens were shown

For patients with peripheral nerve damage with symptoms such as numbness in the hands and feet, neurotrophic drugs can be used in subsequent treatment cycles. Scalp cooling devices can be used to improve the chemotherapy-induced alopecia.

At the end of each course, the adverse effects of chemotherapy were assessed. Toxicities of chemotherapy drugs including hematopoietic toxicity (anemia, leucopenia, neutropenia, and thrombocytopenia), hepatic function, renal function, cardiac function, gastrointestinal toxicity (vomiting and diarrhea), hair loss, and numbness of hands and feet were divided into 4 levels (I–IV) according to the Common Terminology Criteria for Adverse Events [24]. Adverse reactions, such as vomiting and diarrhea, were treated symptomatically.

### Genotyping for the GSTP1 gene

Genomic DNA was extracted from whole blood samples using a QIAamp DNA Blood Mini Kit (Qiagen GmbH, North Rhine-Westphalia, Germany), according to the protocol provided. A NanoDrop2000 Spectrophotometer (Thermo Scientific) was used to determine the concentration and purity of DNA. The genotype of *GSTP1* (Ile105Val, rs1695) was established using Sanger sequencing. The primer sequences and the PCR enzymes were provided by SINOMD Gene Detection Technology Co., Ltd. (Beijing, China). The target fragments were amplified using polymerase chain reaction (PCR): initial denaturation at 95℃ for 3 min, followed by 45 cycles of denaturation at 94℃ for 15 s, annealing at 63℃ for 1 min, and extension at 72℃ for 1 min. ExoSap-It (ABI PCR Product Cleanup Reagent) was used to purify PCR products. ABI Terminator v3.1 Cycle Sequencing kit was used to detect sequences, which were analyzed with Sequencing Analysis v5.4 (Life Technologies, CA, USA) on ABI 3500 Dx Genetic Analyzer.

### Data collection and statistical analysis

Clinical information, including age, gender, histopathological type, TNM stage, tumor grade, molecular subtype, chemotherapy regimen, and toxicity of chemotherapy drugs, was collected. SPSS statistical software version 21.0 (IBM Inc., State of New York, USA) was used for data analysis. The Hardy–Weinberg equilibrium (HWE) of *GSTP1* genotypes was assessed using the *χ*^2^ test. Fisher’s exact test was used to assess the relationship between *GSTP1* variant status and responsiveness and toxicity. A value of *P* < 0.05 was considered statistically significant.

## Results

### Population characteristics

A total of 142 breast cancer patients were subjected in this study. There were 16 (11.3%) patients under the age of 35, 60 (42.2%) patients between the ages of 35 and 50, and 66 (46.5%) patients beyond the age of 50. There were 61 (43.0%) patients in the T0–T2 stages and 81 (57.0%) patients in T3–T4 stages, 43 (30.3%) patients in the N0–N1 stages, and 99 (69.7%) patients in the N2–N3 stages. There were 108 (76.1%) patients in clinical stages I–III and 34 (23.9%) patients in clinical stage IV. The numbers of luminal A, luminal B, HER2 + , and TNBC patients were 10 (7.0%), 77 (54.2%), 33 (23.2%), and 22 (15.5%), respectively (Table 1).

### GSTP1 gene polymorphism frequency in the study patients

*GSTP1* c.313A > G genotyping was performed on all participants in this investigation. The numbers of *GSTP1* c.313A > G A/A, A/G, and G/G genotypes were 94 (66.2%), 45 (31.7%), and 3 (2.1%), respectively. The numbers of *GSTP1* c.313A > G A and G allele was 233 (82.0%) and 51 (18.0%), respectively. The genotypic distribution of *GSTP1* c.313A > G in the participants was consistent with the Hardy–Weinberg equilibrium (*χ*^2^ = 0.809, *P* = 0.368) (Table 2).

**Table 2:** GSTP1 gene polymorphism frequency in the study patients

Genotypes/allele	Number	Percentage (%)
Genotypes		
A/A	94	66.2
A/G	45	31.7
G/G	3	2.1
Allele		
A	233	82.0
G	51	18.0
HWE (*χ*^2^, *P*)	*χ*^2^ = 0.809, *P* = 0.368	

*HWE* Hardy–Weinberg equilibrium; *P* < 0.05 was considered statistically significant

### Adverse events

In this study, 142 patients experienced adverse reactions to chemotherapy drugs. In terms of hematological toxicity, there were 57 cases (40.1%) with leucopenia grade I/II and 71 cases (50.0%) with leucopenia grade III/IV, 69 cases (48.6%) with neutropenia grade I/II, 49 cases (34.5%) with neutropenia grade III/IV, 87 cases (61.3%) with anemia grade I/II, 11 cases (7.7%) with anemia grade III/IV, 37 cases (26.1%) with thrombocytopenia grade I/II, and 27 cases (19.0%) with thrombocytopenia grade III/IV. There were 74 (52.1%), 5 (3.5%), and 1 (0.7%) patients with hepatic function, renal function, and cardiac function toxicity, respectively. In terms of gastrointestinal toxicity, there were 123 cases (86.6%) with vomiting grade I/II and 5 cases (3.5%) with diarrhea grade I/II. In addition, there were 142 cases (100.0%) with hair loss grade I/II and 91 cases (64.1%) with numbness of hands and feet grade I/II. The proportions of chemotherapy toxicities in *GSTP1* c.313A > G wild-type and *GSTP1* c.313A > G mutant patients are shown in Table 3.

**Table 3:** Frequency of all grade toxicities in patients

Toxicities	Total (*n* = 142)		*GSTP1* c.313A > G wild-type (*n* = 94)		*GSTP1* c.313A > G mutation (*n* = 48)	
	Grades I/II	Grades III/IV	Grades I/II	Grades III/IV	Grades I/II	Grades III/IV
Hematological toxicity						
Leucopenia	57 (40.1%)	71 (50.0%)	40 (42.6%)	44 (46.8%)	17 (35.4%)	27 (56.3%)
Neutropenia	69 (48.6%)	49 (34.5%)	41 (43.6%)	32 (34.0%)	28 (58.3%)	17 (35.4%)
Anemia	87 (61.3%)	11 (7.7%)	57 (60.6%)	7 (7.4%)	30 (62.5%)	4 (8.3%)
Thrombocytopenia	37 (26.1%)	27 (19.0%)	21 (22.3%)	17 (18.1%)	16 (33.3%)	10 (20.8%)
Hepatic function	71 (50.0%)	3 (2.1%)	46 (48.9%)	1 (1.1%)	25 (52.1%)	2 (4.2%)
Renal function	5 (3.5%)	0 (0)	3 (3.2%)	0 (0)	2 (4.2%)	0 (0)
Cardiac function	1 (0.7%)	0 (0)	1 (1.1%)	0 (0)	0 (0)	0 (0)
Gastrointestinal toxicity						
Vomiting	123 (86.6%)	0 (0)	81 (86.2%)	0 (0)	42 (87.5%)	0 (0)
Diarrhea	5 (3.5%)	0 (0)	2 (2.1%)	0 (0)	3 (6.3%)	0 (0)
Hair loss	142 (100.0%)	0 (0)	94 (100.0%)	0 (0)	48 (100.0%)	0 (0)
Numbness of hands and feet	91 (64.1%)	0 (0)	60 (63.8%)	0 (0)	31 (64.6%)	0 (0)

### *Association between GSTP1 c.313A* > *G genotypes and toxicities*

The association between *GSTP1* c.313A > G genotypes and the grade of adverse reactions of chemotherapy is shown in Table 4. In terms of hematological toxicity caused by chemotherapy, there were no statistically significant differences in the proportions of leucopenia, anemia, and thrombocytopenia in patients with A/G, G/G, and A/G + G/G genotypes compared to patients with the A/A genotype (all *P* > 0.05). However, the proportion of neutropenia in patients with the A/G genotype (grade I/II 55.6% and grade III/IV 37.8%) was significantly higher than that in A/A genotype patients (grade I/II 43.6% and grade III/IV 34.0%) (*χ*^2^ = 5.604, *P* = 0.050), and A/G + G/G genotype (grade I/II 58.3% and grade III/IV 35.4%) was also higher than that in A/A genotype patients (*χ*^2^ = 6.586, *P* = 0.035). Furthermore, there were no statistically significant differences in the proportion of abnormal hepatic function, renal function, and cardiac function in patients with A/G, G/G, and A/G + G/G genotypes compared to patients with the A/A genotype (all *P* > 0.05). In terms of gastrointestinal toxicity caused by chemotherapy, there were no statistically significant differences in the proportion of vomiting and diarrhea in patients with A/G, G/G, and A/G + G/G genotypes compared to patients with the A/A genotype (all *P* > 0.05) (Table 4).

**Table 4:** Association between polymorphism of GSTP1 genes and toxicities of chemotherapy

Toxicities	A/A (*n* = 94)	A/G (*n* = 45)	G/G (*n* = 3)	A/G + G/G (*n* = 48)
Hematological toxicity				
Leucopenia				
No toxicities	10(10.6%)	4(8.9%)	0(0)	4(8.3%)
Grade I/II	40(42.6%)	16(35.6%)	1(33.3%)	17(35.4%)
Grade III/IV	44(46.8%)	25(55.6%)	2(66.7%)	27(56.3%)
*P* value^*^		0.652	1.000	0.574
Neutropenia				
No toxicities	21(22.3%)	3(6.7%)	0(0)	3(6.3%)
Grade I/II	41(43.6%)	25(55.6%)	3(100.0%)	28(58.3%)
Grade III/IV	32(34.0%)	17(37.8%)	0(0)	17(35.4%)
*P* value^*^		0.050	0.311	0.035
Anemia				
No toxicities	30(31.9%)	12(26.7%)	2(66.7%)	14(29.2%)
Grade I/II	57(60.6%)	29(64.4%)	1(33.3%)	30(62.5%)
Grade III/IV	7(7.4%)	4(8.9%)	0(0)	4(8.3%)
*P* value^*^		0.810	0.432	0.961
Thrombocytopenia				
No toxicities	56(59.6%)	19(42.2%)	3(100.0%)	22(45.8%)
Grade I/II	21(22.3%)	16(35.6%)	0(0)	16(33.3%)
Grade III/IV	17(18.1%)	10(22.2%)	0(0)	10(20.8%)
*P* value^*^		0.145	0.756	0.265
Hepatic function				
No toxicities	47(50.0%)	20(44.4%)	1(33.3%)	21(43.8%)
Grade I/II	46(48.9%)	23(51.1%)	2(66.7%)	25(52.1%)
Grade III/IV	1(1.1%)	2(4.4%)	0(0)	2(4.2%)
*P* value^*^		0.386	1.000	0.402
Renal function				
No toxicities	91(96.8%)	43(95.6%)	3(100.0%)	46(95.8%)
Grade I/II	3(3.2%)	2(4.4%)	0(0)	2(4.2%)
Grade III/IV	0(0)	0(0)	0(0)	0(0)
*P* value^#^		0.659	1.000	1.000
Cardiac function				
No toxicities	93(98.9%)	45(100.0%)	3(100.0%)	48(100.0%)
Grade I/II	1(1.1%)	0(0)	0(0)	0(0)
Grade III/IV	0(0)	0(0)	0(0)	0(0)
*P* value^#^		1.000	1.000	1.000
Gastrointestinal toxicity				
Vomiting				
No toxicities	13(13.8%)	6(13.3%)	0(0)	6(12.5%)
Grade I/II	81(86.2%)	39(86.7%)	3(100.0%)	42(87.5%)
Grade III/IV	0(0)	0(0)	0(0)	0(0)
*P* value^#^		1.000	1.000	1.000
Diarrhea				
No toxicities	92(97.9%)	43(95.6%)	2(66.7%)	45(93.8%)
Grade I/II	2(2.1%)	2(4.4%)	1(33.3%)	3(6.2%)
Grade III/IV	0(0)	0(0)	0(0)	0(0)
*P* value^#^		0.595	0.091	0.336
Hair loss				
No toxicities	0(0)	0(0)	0(0)	0(0)
Grade I/II	94(100.0%)	45(100.0%)	3(100.0%)	48(100.0%)
Grade III/IV	0(0)	0(0)	0(0)	0(0)
*P* value^#^		-	-	-
Numbness of hands and feet				
No toxicities	34(36.2%)	16(35.6%)	1(33.3%)	17(35.4%)
Grade I/II	60(63.8%)	29(64.4%)	2(66.7%)	31(64.6%)
Grade III/IV	0(0)	0(0)	0(0)	0(0)
*P* value^#^		1.000	1.000	1.000

^*^Whether toxicities and the grade of toxicities in patients with mutant genotype vs. wild-type, respectively

^#^Toxicities or no in patients with mutant genotype vs. wild-type, respectively

The logistic regression analysis was performed to determine independent variables associated with neutropenia. The variables included age, menopausal status, T stage, N stage, clinical stage, molecular type, and chemotherapy regimen/dose (classified by chemotherapy, dose of anthracycline, and paclitaxel). Of these patients, 5 patients were excluded from the analysis because used anthracycline or paclitaxel alone (TCbH, TCb, and EC regimen) and the number of cases was small. The results indicated that *GSTP1* c.313A > G mutation (A/G + G/G vs. A/A genotype) (age-, menopause-, T-stage, N-stage, clinical stage-, molecular subtype-, and chemotherapy regimen/dose-adjusted OR 4.273, 95% CI 1.141–16.000, *P* = 0.031) was an independent variable associated with neutropenia. No correlation was found between toxicity and patients’ age, tumor staging, molecular subtype, menopause status, and chemotherapy regimen/dose (Table 5).

**Table 5:** Logistic regression analysis of variables associated with neutropenia

Variables	Unadjusted values		Adjusted values	
	*P* value	OR (95% CI)	*P* value	Adjusted OR (95% CI)
Age (≤ 50/ > 50)	0.163	0.515 (0.202–1.309)	0.127	0.252 (0.043–1.483)
Menopause (no/yes)	0.733	0.853 (0.341–2.131)	0.324	2.446 (0.414–14.440)
T-stage (T3-T4/T0-T2)	0.965	1.020 (0.413–2.520)	0.593	1.316 (0.481–3.597)
N-stage (N2-N3/N0-N1)	0.313	0.578 (0.199–1.678)	0.320	0.553 (0.172–1.776)
Clinical stage (IV/I–III)	0.911	1.064 (0.360–3.142)	0.860	1.113 (0.340–3.637)
Molecular subtype (TNBC/non-TNBC)	0.817	1.168 (0.313–4.367)	0.450	1.747 (0.410–7.442)
Chemotherapy regimen/dose ((docetaxel 75 mg/m^2^ and epirubicin 75 mg/m^2^)/(docetaxel 90 mg/m^2^ and epirubicin 90 mg/m^2^))	0.197	2.014 (0.695–5.835)	0.124	2.454 (0.782–7.697)
*GSTP1* c.313A > G (A/G + G/G vs. A/A)	0.031	4.038 (1.132–14.395)	0.031	4.273 (1.141–16.000)

*TNBC* Triple-negative breast cancer

## Discussion

Breast cancer is one of the most common malignant tumors in women [1]. Adjuvant chemotherapy is a crucial part of the comprehensive treatment of breast cancer. Chemotherapeutic drugs, on the other hand, destroy a huge number of bone marrow cells as well as tumor cells, due to a lack of targeting, resulting in bone marrow suppression and hematologic adverse reactions [25]. Clinically, patients receiving the same dose of the same chemotherapeutic drug may experience distinct adverse reactions, which are difficult to explain without considering patients’ clinical factors (such as age, tumor stage and grade, and hormone receptor status) and environmental factors [26]. As gene sequencing technology advances and the need for precision therapy grows, clinicians and researchers are paying more and more attention to the role of pharmacogenetics in breast cancer chemotherapy [27].

GSTP1 is a member of the GST family, which is involved in catalyzing the formation of glutathione disulfide bonds for the protection of cells against oxidative stress. The *GSTP1* rs1695 (c.313A > G, Ile105Val) polymorphism may influence GSTP1 enzyme activity, which is linked to chemotherapy drug detoxification and tumor cell sensitivity [28–30]. The *GSTP1* rs1695 polymorphism has been linked to higher toxicity in several studies [31–33]. On the contrary, another study found that febrile neutropenia was prevalent among patients with the A/A genotype [23]. According to research, *GSTP1* Ile105Val mutant enzymes induce high expression of intracellular defense proteins, which protect cells from chemotherapy drug toxicity by decreasing and inhibiting JNK (C-Jun NH2-terminal kinase) [34]. These discrepancies could be attributable to ethnic disparities, sample size, administration method, and the usage of multiple drugs. Furthermore, investigations have shown that certain genes, signaling pathways, and lncRNAs play a role in tumorigenesis, drug response, and metastasis [35–37]. All of these provide us new ideas to further study the adverse reactions and prognosis of chemotherapy drugs, as well as identify the reasons for the inconsistent results.

There have been few research on the connection between *GSTP1* polymorphism and anthracycline-/paclitaxel-based chemotherapy toxicity. The *GSTP1* c.313 A > G mutation was found to be an independent risk factor for neutropenia hematotoxicity induced by anthracycline-/paclitaxel-based chemotherapy in breast cancer patients. Our findings are consistent with some of the findings of previously reported studies [22, 38, 39]. On the contrary, in a Japanese population, breast cancer patients treated with epirubicin and cyclophosphamide, as well as those with the *GSTP1* c.313A > G A/A genotype were more likely to develop febrile neutropenia [23]. In a North American population, patients with the *GSTP1* c.313A > G A/A genotype had a lower incidence of grade III and IV neutropenia than those with the *GSTP1* c.313A > G G allele [40]. A clinical trial showed that patients with the GSTP1*A (Ile105/Ala114)/*B (Val105/Ala114) genotype may experience increased hematologic toxicity when treated with docetaxel chemotherapy [41]. Furthermore, another study found that *GSTP1* c.313A > G was not linked to neutropenia in patients receiving chemotherapy with cyclophosphamide (CP), methotrexate (MTX), and 5-fluorouracil (5-FU) (CMF treatment) or a combination of 5-FU, anthracycline-based chemotherapy (adriamycin or its analog epirubicin), and CP (FAC/FEC treatment) regimens [42]. In addition, the relationship between *GSTP1* gene polymorphism and adverse reactions related to chemotherapy drugs may be inconsistent in different cancer types and different treatment regimens. For example, Deng et al. found that colorectal cancer patients with *GSTP1* c.313A > G mutation who received treatment with fluoropyrimidines and oxaliplatin had an increased risk of severe vomiting (grade III/IV), but there was no relationship between the polymorphism and neutropenia. And it showed that *GSTP1* c.313A > G mutation may be an independent risk factor for severe vomiting induced by chemotherapeutic drugs [43].

Furthermore, in this investigation, there was no correlation between toxicity effect and patients’ age, tumor staging, molecular subtype, and menopause status in this study. A study showed that grade III or IV toxicities were more frequent in elderly patients [44]. Another study showed that elderly and younger patients had a similar frequency and number of toxicities [45]. The clinical stage of breast cancer may be related to the degree of toxicity of chemotherapy [46]. There are currently no investigations on the link between menopausal status and anthracycline- and/or paclitaxel-related toxicity in patients with breast cancer.

This is the first study in the Hakka population to look at the link between *GSTP1* c.313A > G genotypes and clinical toxicity of anthracycline-/paclitaxel-based chemotherapy in breast cancer patients. Nevertheless, there are some limitations to this study that should be noted. First, the number of subjects in this research is relatively small, leading to some deviations in the results. Second, we only investigated one single-nucleotide polymorphism (SNP) of *GSTP1* linked to anthracycline-/paclitaxel-related toxicity, and the status of additional SNP sites in these patients is unknown. As a result, one of the next steps will be to conduct additional research with larger sample size and to conduct a comprehensive analysis of the *GSTP1* gene.

In conclusion, the results of this study indicate that the *GSTP1* c.313A > G mutation is an independent risk factor for neutropenia hematotoxicity induced by anthracycline-/paclitaxel-based chemotherapy in breast cancer patients. This is the first study of its kind among the Hakka population. Research on the relationship between drug metabolism gene polymorphism and chemotherapy toxicity can predict and avoid toxic reactions, which can help breast cancer patients improve their quality of life. However, genetic screening only identify those groups of patients who are likely to suffer from adverse effects. Reducing the degree of distress related to chemotherapy drugs requires scientific and detailed pre-chemotherapy care programs, timely and adequate communication between patients and doctors, and effective coping strategies [47].

## Data Availability

The datasets used and analyzed during the current study available from the corresponding author on reasonable request.
